# An Outline of Multi-Sensor Fusion Methods for Mobile Agents Indoor Navigation

**DOI:** 10.3390/s21051605

**Published:** 2021-02-25

**Authors:** Yuanhao Qu, Minghao Yang, Jiaqing Zhang, Wu Xie, Baohua Qiang, Jinlong Chen

**Affiliations:** 1 Research Center for Brain-inspired Intelligence (BII), Institute of Automation, Chinese Academy of Sciences (CASIA), Beijing 100190, China; 1803403006@mails.guet.cn; 2 School of Computer and Information Security, Guilin University of Electronic Technology, Guilin 541004, China; 54zhang.peng@163.com (J.Z.); xiesixchannels@126.com (W.X.); qiangbh@guet.edu.cn (B.Q.); 7259@163.com (J.C.)

**Keywords:** mobile agent, multi-sensor fusion, multi-modal dataset, SLAM (simultaneous localization and mapping)

## Abstract

Indoor autonomous navigation refers to the perception and exploration abilities of mobile agents in unknown indoor environments with the help of various sensors. It is the basic and one of the most important functions of mobile agents. In spite of the high performance of the single-sensor navigation method, multi-sensor fusion methods still potentially improve the perception and navigation abilities of mobile agents. This work summarizes the multi-sensor fusion methods for mobile agents’ navigation by: (1) analyzing and comparing the advantages and disadvantages of a single sensor in the task of navigation; (2) introducing the mainstream technologies of multi-sensor fusion methods, including various combinations of sensors and several widely recognized multi-modal sensor datasets. Finally, we discuss the possible technique trends of multi-sensor fusion methods, especially its technique challenges in practical navigation environments.

## 1. Introduction

As early as the 1960s, there were science fiction films and TV works depicting the autonomous navigation of mobile agents: intelligent robots that are able to travel freely in indoors such as in offices, factories, shopping malls, and hospitals and help people in work, production, play, and study. In these scenarios, autonomous navigation ability is the basic and one of the most important functions of mobile agents.

Autonomous indoor navigation of mobile agents refers to the abilities of autonomous localization and map construction in dynamic scenes [1]. This technology is based on the collection, analysis, and perception of environmental information, and carries out real-time localization and route planning by constructing a map. Using the data acquired by sensors to perceive the environment is the key technique related to mobile agent navigation. Historically, various sensors have been adopted for mobile agent navigation, such as cameras [2], light detection and ranging (LiDAR) [3], inertial measurement units (IMU) [4], ultra-wide band (UWB) [5], Wi-Fi [6], Bluetooth [7], ZigBee [8], infrared [9], ultrasonic [10], etc. According to the different principles and usages of these sensors, some scholars divided autonomous navigation into two categories: single-sensor navigation methods and multi-sensor fusion navigation methods [11].

In the single sensor navigation methods, the agents decide their own navigation states in the environment depending on a single sensor [12], among which cameras and LiDAR are widely used. A single sensor has specific advantages and limitations in navigation: for example, the visual sensor has the advantages of low price and various mature algorithms provided by researchers. However, the vision perception accuracies are easily influenced by the environments’ changes in illumination [13]. Correspondingly, LiDAR data have the advantage of high frequency. However, LiDAR data’s resolution usually requires improvement and the content information is not intuitively presented in it [13]. Compared to single-sensor agent navigation, multi-sensor fusion methods improve the localization accuracy and robustness in the task of navigation by collecting and fusing the environmental information from different types of sensors [11]. In recent years, research on multi-sensor fusion methods for agent navigation has become an important trend.

The goal of this paper is not to present a complete summary of mobile agents’ navigation, but to focus on the discussions of multiple sensors’ fusion methods in mobile agents’ indoor navigation. In spite of several recent publications on related work [14,15], this work is obviously different from them, in that we focus on the multi-sensor fusion methods rather than a discussion of positioning [14], mapping, and way finding [15]. Namely, we focus on the discussion of sensors’ functionalities and the fusion methods of them in navigation rather than pure algorithms or the navigation task.

According to the event where and when fusions were processed, fusion could happen at the data (feature) level, model level, and decision level, respectively [16]. According to the calculation method, the fusion can be divided into rule-based and statistic (machine learning)-based fusion [17]. Considering the relativity of different channels, some literature divided their relationships into three categories: complementary, mutual exclusion, and redundancy [18]. In this work, considering the fact that the sensors play key roles in the perception and decision making in navigation, we divide the multi-sensor fusion models into two types: one dominant sensor combined with assisting sensors [19], and multiple sensors assisting each other without a dominant sensor [20]. We believe that this strategy benefits the outline of each sensor, and helps to learn sensors’ advantages and disadvantages in the fusion procedure. To this end, we focus on the fusion methods of different possible sensors in navigation, including traditional classical fusion methods, but also the new methods introduced in recent years, such as deep learning and reinforcement learning on sensor fusion. In each section, we start with the introduction of traditional methods, and then gradually transition to the new developments in this area in recent years.

In addition, quite a few multi-sensor fusion methods are proposed by the experiments, which are based on both indoor and outdoor environments, and a large portion of fusion methods have high versatility in various environments. Due to the stability and safety of indoor environments, compared with that of outdoors, we believe that some of these methods still have the same potential in indoor navigation. In this way, a few multiple fusion methods originally proposed in outdoor environments are also included in our discussions, since the fusion methods are worth referencing.

The remainder of this paper is organized as follows: we first briefly review some widely used sensors in Section 2; the main multi-sensor fusion methods for agent navigation and some well-known multi-modal datasets are introduced in Section 3 and Section 4; and the discussions and possible technique trends of multi-sensor fusion methods are given in Section 5. Finally, Section 6 concludes the whole work.

## 2. Single Sensor Based Navigation

There are multiple kinds of sensors have been used for mobile navigation, including visual sensors [2], LiDAR [3], IMU [4], and UWB devices [5]. In addition to these four widely-used types of sensors, there are also some other sensors, such as Wi-Fi [6], Bluetooth [7], etc.

### 2.1. Visual Sensors

There are three types of cameras that are widely used in navigation tasks: the monocular camera [2], the stereo camera [21], and the RGB-D camera [22]. Monocular vision navigation tasks are divided into three categories: the feature points matching method, the direct method, and the semi-direct method. One of the most well-known point matching methods is oriented fast and rotated brief simultaneous localization and mapping (SLAM) (ORB-SLAM) [2], which is developed based on parallel tracking and mapping for small augmented reality (AR) workspaces (PTAM) [23]. ORB-SLAM extracts the image ORB features for calculation and optimization in navigation. Because the calculation speed of ORB features is fast, and it is not likely to be affected by noise and image transformation to a certain extent, ORB-SLAM is a good optimization strategy for navigation [21]. A typical representative of the direct method is large-scale direct monocular SLAM (LSD-SLAM) [24], which estimates the motion of the camera based on the gradient of the pixels, so there is no need to calculate feature points, and it can also construct a semi-dense scene map. Although the direct method reduces the calculation of feature points compared to the feature point method, the direct method is more sensitive to camera exposure and is more likely to lose information during rapid movement. The representative of the semi-direct method is semi-direct visual odometry (SVO) [25], which is a combination of the feature point method and the direct method. It is mainly used in unmanned aerial vehicles (UAVs) and achieves faster operating speeds. In general, the feature point method is robust and flexible [26].

Stereo cameras are mostly divided into binocular cameras and trinocular cameras. It is usually equipped with more than two lenses. Multiple lenses simultaneously shoot the same scene from different angles, and then perform complex feature matching, so as to more accurately restore 3D information of the scene [26]. Navigation methods with multi-eye cameras have been widely used in mobile robot localization and navigation, obstacle avoidance and map construction. At present, vision localization systems such as ORB-SLAM 2 [21] and real-time appearance-based mapping (RTAB-MAP) [22] provide solutions adopted for binocular sensors. However, the stereo vision system meets challenges in poor feature point environments, which restricts the application prospect of stereo vision [13]. 

The RGB-D camera is equipped with an ordinary camera and an infrared transmitter/receiver [27]. It is able to obtain the depth of the environment. The emergence of RGB-D cameras simplifies the structure of the visual localization system to a certain extent. At present, there are a few mature RGB-D visual navigation solutions, such as RGB-D SLAM V2 [27], dense visual odometry-SLAM (DVO-SLAM) [28], and RTAB-MAP [22], etc. At present, the main RGB-D camera products on the market are Kinect V2, RealSense SR300, Xtion2, etc., with relatively low prices. However, RGB-D cameras still have the unavoidable noises caused by sunlight interference or backlight. In spite of the advantage of obtaining the objects’ appearance information in scene, the visual sensors themselves are not ideal for in-depth information perception.

### 2.2. LiDAR

LiDAR uses laser technology to measure distance. The agent can determine its relative localization in the environment based on the distance information collected by LiDAR [13].

In general, LiDAR navigation methods are divided into two styles: 2D LiDAR and 3D LiDAR. The 2D LiDAR navigation method has been widely discussed: grid mapping (GMapping) [29], Hector [3], etc. Although 2D LiDAR navigation is able to work in real time, it lacks the height information of objects and has difficulty in constructing a 3D map. Three-dimensional LiDAR navigation is able to collect depth information from different heights, and can complete real-time imaging to restore the shape of objects. Representative solutions of 3D LiDAR navigation include implicit moving least squares-SLAM (IMLS-SLAM) [30] and LiDAR odometry and mapping (LOAM) [31], etc. At present, 3D LiDAR navigation has become the research mainstream, and has been widely used in autonomous driving and robotics [1].

In terms of data processing, LiDAR navigation methods are mainly divided into two types: filtering and optimization. The representative of the filtering methods is GMapping [29], which is a laser localization strategy based on the Rao–Blackwellized particle filter (RBPF). This algorithm obtains high localization accuracy in a small scene map. Due to the use of particle filter, this method is not suitable for the situation when there is a large scene or too many particles. The representative of the optimization methods is Cartographer [32]. Low-end equipment can also perform well using the optimization method and the cumulative errors of Cartographer are less than GMapping [31].

LiDAR has a wide detection range and is not easily affected by light, so it has strong adaptability to the environment. Moreover, laser ranging has a good performance regarding obstacle avoidance, and relative localization when facing towards objects. However, compared with visual sensors, LiDAR cannot obtain semantic information and the color and boundary of the object.

### 2.3. Inertial Measurement Unit

The inertial measurement unit (IMU) is a device that measures the angular velocity and acceleration of an object’s posture. It usually consists of a three-axis gyroscope and a three-axis accelerometer. Some IMUs even contain nine axes [33], in which a three-axis geo-magnetometer is added to the six-axis IMU.

The IMU is fixed on the mobile agent and obtains the real-time angular velocity and acceleration of the movement at a high frequency. Through the integration of angular velocity and acceleration, the angle and the distance of the motivation within a certain period of time can be calculated [34]. However, the IMU can only obtain the motion posture of itself, and cannot collect environmental information. In practice, multiple measurements with the IMU can easily cause cumulative errors, and the obtained data cannot be used for localization for a long time [11]. Therefore, IMU-based navigation methods are mostly combined with other sensors, such as LiDAR-inertial odometry (LIO) [35], where the IMU is combined with LiDAR, and multi-state constraint Kalman filter (MSCKF), where the IMU is combined with a visual sensor [36].

Nowadays, the IMU is mostly integrated onto the agent platform in the form of a micro-electro-mechanical system (MEMS). Compared with LiDAR, the MEMS-IMU is cheaper, smaller, and lighter. Most commercial indoor robots are integrated with the MEMS-IMU, such as Turtlebot, RB-1 BASE, etc.

### 2.4. Ultra-Wide Band Localizationing System

Ultra-wide band (UWB) technology transmits data by sending and receiving extremely narrow pulses of nanoseconds or less, and thus has a bandwidth in the order of GHz [37]. The UWB localization system needs the arrangement of more than four localization base stations with known coordinates, and the agent needs to be equipped with pulse receiving and returning equipment. The equipment transmits pulses at a certain frequency to measure the distance between the base stations and the agent [38]. Because of the limitations of equipment installation, the UWB localization system is usually used in relatively empty indoors, such as factories and warehouses.

UWB ranging methods include time of flight (TOF) [39], angle of arrival (AOA) [40], time of arrival (TOA) [40], and time difference of arrival (TDOA) [37]. Using different methods will have a certain impact on the accuracy and real-time performance, but the overall accuracy can reach a high level. The most advanced UWB localization equipment has an error within 10 cm [37].

The UWB localization system has the advantages of low power consumption, high localization accuracy, and high transmission rate. Moreover, the UWB localization does not rely on the calculation of the agent, which helps to reduce the calculation cost of the localization. However, the UWB localization strategy only determines the localization of the mobile agent, but cannot build a map separately. Therefore, autonomous navigation with the UWB system usually needs to be combined with other sensors. In addition, the equipment usually needs to be pre-installed before working, which reduces the portability and the usage of UWB system.

### 2.5. Others and a View

In addition to these four main kinds of sensors, other sensors including Wi-Fi, Bluetooth, ZigBee, infrared sensors, and ultrasonic sensors, etc., are also widely used in spatial localization or distance measurement. In special indoor scenes, these sensors have special applications, such as in situations where visually impaired people operate agents [41], and low-power wireless signals are involved [42,43], etc. However, with similar functionality to the UWB, these wireless sensors do not provide the high localization accuracy and anti-interference ability found in UWB devices [31].

Table 1 presents a list of single-sensor navigation methods, including their classical application environments, advantages, and disadvantages. In general, the visual sensors have high performances in constructing visual maps, but their localization accuracies need to be improved. LiDAR methods have high measurement accuracy, but the context information is lost in their presentation. UWB devices provide high localization accuracy, but they are only suitable for in a specific range. The IMU is a good tool to obtain the state of motion in a short time. However, it does not provide environmental information and it has the disadvantage of unavoidable cumulative errors in a long-term measurement. The IMU is mostly used as an auxiliary tool in autonomous navigation.

## 3. Multi-Sensors Fusion Navigation Methods

In spite of the high performance of single-sensor navigation, single-sensor navigation methods still have their limitations. It has been reported that multi-sensor fusion methods potentially improve the perception and navigation ability of mobile agents: first, multi-sensor fusion enriches the modes of information acquisition and enhances the ability to collect information in a specific environment; second, multi-sensor fusion organically combines different modal information according to the sensors’ internal relationship, which helps to improve the localization accuracy. Now, the research on multi-sensor fusion navigation has become the mainstream.

According to the fusion strategy, the fusion models are divided into two types: one dominant sensor combined with assisting sensors [19], and multiple sensors assisting each other without dominant sensors [20]. In this section, we mainly discuss the characteristics of different kinds of multi-sensor fusion methods for indoor navigation tasks, and introduce some representative multi-sensor fusion programs.

### 3.1. Visual Sensor-Dominant Navigation

The visual sensors provide context information relating to the environment and construct a visual map. However, they are easily affected by illumination changes. In order to overcome these deficiencies, other sensors, such as LiDAR, IMU, and UWB, are used to improve the visual navigation.

#### 3.1.1. Vision Combined with Depth Information

Although RGB images and depth data are different modalities, the RGB-D camera is able to collect both of them simultaneously [27]. The point cloud provided by the RGB-D camera contains RGB data and 3D coordinates, but the relative motion state of the agent cannot be obtained by visual sensors alone [44]. In order to realize the autonomous navigation, it is also necessary to perform motion pose estimation for the mobile agent [13]. Motion pose estimation helps to restore the trajectory of the agent, so as to construct a map by determining the relative localization of the mobile agent.

For the pose estimation strategies for the fusion of RGB and depth information, there are three kinds of methods: feature point matching, the optical flow method, and the direct method. In addition, RGB-D visual semantic mapping based on deep learning is also a new kind of fusion trend.

(1) Feature Point Matching

Feature point motion estimation is widely used in traditional visual localization methods, such as ORB-SLAM [2] and scale-invariant feature transform (SIFT) [45], etc. In the case of RGB and depth data fusion processing, because the images collected by RGB-D cameras have spatial location information, the feature points of RGB-D images are actually 3D points with spatial information. The motion estimation of 3D points is dealt with using perspective-n-point (PnP) [46] or iterative close point (ICP) [47]. Several approaches have been proposed to solve PnP and ICP [48], including direct linear transformation (DLT) [49] and singular value decomposition (SVD) [50].

Equation (1) presents a mathematical description for DLT, where a point $P={\left(X,Y,Z,1\right)}^{T}$ is projected to the matching point $x={\left(u_{1},v_{1},1\right)}^{T},$ and s is the scale factor. In addition, the 3×4 matrix between P and x are rotation and translation parameters.

(1) $$s\left[\begin{array}{c} u_{1}\\ v_{1}\\ 1 \end{array}\right]=\left[\begin{array}{cccc} t_{1} & t_{2} & t_{3} & t_{4}\\ t_{5} & t_{6} & t_{7} & t_{8}\\ t_{9} & t_{10} & t_{11} & t_{12} \end{array}\right]\left[\begin{array}{c} \begin{array}{c} X\\ Y\\ Z\\ 1 \end{array} \end{array}\right]$$

With simplifications to Equation (1), the transformation matrix can be solved by substituting matching point pairs from Equation (2) with common equation solving methods, such as the Gauss-Newton method.

(2) $$\left[\begin{array}{c} \begin{array}{ccc} P_{1}^{T} & 0 & -u_{1}P_{1}^{T}\\ 0 & P_{1}^{T} & -u_{1}P_{1}^{T}\\ \vdots & \vdots & \vdots \end{array}\\ \begin{array}{ccc} P_{N}^{T} & 0 & -u_{N}P_{N}^{T} \end{array}\\ \begin{array}{ccc} 0 & P_{N}^{T} & -u_{N}P_{N}^{T} \end{array} \end{array}\right]\left[\begin{array}{c} t_{1}\\ t_{2}\\ t_{3} \end{array}\right]=0$$

The SVD method of ICP is generally divided into three steps:

Step 1. Calculate the centroid $p,\;p^{\prime }$ of the two sets of points, and then calculate the de-centroid coordinates of each point:

(3) $$q_{i}=p_{i}-p,\;q_{i}^{\prime }=p_{i}^{\prime }-p^{\prime }$$

Step2. Calculate the rotation matrix:

(4) $$R^{*}=\arg\underset{R}{\min}\frac{1}{2}\overset{n}{\underset{i=1}{\sum }}∥q_{i}-Rq_{i}^{\prime }∥{}^{2}$$

Step3. Calculate t according to R in Step 2, where *R*, *t* is the Euclidean transformation of ICP:

(5) $$t^{*}=p-Rp^{\prime }$$

The PnP method is usually adopted in a 3D to 2D point register, while ICP often used in a 2D/2D or 3D/3D point register. However, sometimes the depth information obtained by RGB-D camera contains larger errors; in this case, PnP and ICP are combined in RGB-D point register where PnP solution is adopted firstly to select the outliers and ICP is continually used to improve accuracies for the point register [51]. This hybrid PnP method helps to compute the pose change between two camera frames when depth values are rather noisy, thereby improving the accuracy of pose estimation.

There are cases that the feature point method is dependent on the quality of feature points’ selection, and it cannot work well when there are too few feature points [13]. In practical calculation, PnP or ICP problems’ solutions usually rely on linear algebraic methods [52] or nonlinear optimization methods [53]. Usually, feature point methods are time-consuming due to them generating point descriptors and matching, and thus suffer from insufficient real-time demands [24]. Generally speaking, feature point methods are still limited in practical applications, especially in bad environments such as light changes and obstructions.

(2) Optical Flow Method

The optical flow method is based on feature point tracking and matching. The representative is Lucas-Kanade (LK) optical flow [54]. The optical flow method is based on the assumption that the gray level of the same space is constant in each image [55]. This is a strong assumption, which may not be true in practice: when the camera adjusts the exposure parameters, the acquired image will be brighter or darker as a whole, and the gray level of the image will also change. Article [56] proposed using optical flow residuals to highlight the dynamic semantics in the RGB-D point clouds and provide more accurate and efficient dynamic/static segmentation for camera tracking and background reconstruction. This approach achieved accurate and efficient performances in both dynamic and static environments compared to state-of-the-art approaches.

Recently, there are researchers who have adopted deep learning techniques in optical flow. For example, article [57] proposed a convolutional neural network (CNN)-based nonlinear feature embedding optical flow method. CNN-based optical flow helps to improve the quality of optical flow and the accuracy of optical flow pose estimation. However, the deep learning-based optical flow method has the disadvantage of being time-consuming.

(3) Direct Method

The direct method is developed from the optical flow method. It estimates the motion of the camera according to the brightness of pixels, eliminating the calculation of descriptors and feature points [28]. The direct method is divided into three types: sparse, dense, and semi-dense. Compared with the feature point method, which can only construct sparse maps, the direct method can construct dense and semi-dense maps [27].

In the calculation of the direct method, a spatial point with a known localization is required. By using the RGB-D camera, any pixel contains spatial localization information that can be back-projected into 3D space, and then projected to the next image. The information about this spatial point will need to be calculated separately when using monocular or stereo vision systems, but the RGB-D camera can directly obtain it, which simplifies the steps and reduces the calculation. Therefore, the direct method is more suitable for motion estimation used in RGB-D vision systems than monocular and binocular vision.

The direct method can work fast on a low-end computing platform, so it is suitable for occasions with high real-time performance and limited computing resources. However, the direct method belongs to the non-convex optimization algorithm, so it cannot track well when the camera moves in a large scale [28]. In addition, the direct method is also based on the assumption that the gray level is constant in each image. Therefore, it relies on advanced camera equipment to ensure the acquisition image is clear as well as to maintain stable exposure.

The application of RGB-D cameras has greatly changed the way in which the visual sensor obtains the environmental depth information. RGB images and depth data are collected together, which optimizes the space–time synchronization of environmental information collection. In addition, the emergence of the RGB-D camera also promotes the development of point cloud mapping and 3D visualization in more details. However, RGB-D cameras obtain depth information depending on infrared reflection, hollows, and speckle burrs which exist in the depth image because of strong backlight, far or near objects. The RGB-D camera inherits the disadvantage of traditional cameras in that it is sensitive to light.

(4) Semantic SLAM Based on Deep Architectures

Semantic SLAM extends the research content of traditional SLAM, and its research is currently focused on indoor environments. The fusion of RGB images and depth information helps semantic SLAM, mainly embodied in the semantic segmentation of environmental targets, while those based on deep learning are the common methods.

Article [58] proposed an approach to object-class segmentation from multiple RGB-D views using deep learning. It inputs RGB images and depth images into a deep neural network at the same time, and predicts object-class semantics that are consistent from several viewpoints in a semi-supervised way. The semantics predictions of this network can be fused more consistently in semantic keyframe maps than predictions of a network trained on individual views. Article [59] creatively proposed a 3D graph neural network (3DGNN) for RGB-D semantic segmentation. This graph neural network is built on top of points with color and depth extracted from RGBD images. The 3DGNN leverages both the 2D appearance information and 3D geometric relations. It is capable of capturing the long-range dependencies within images, which has been difficult to model in traditional methods. This model achieves good performance on standard RGBD semantic segmentation benchmarks.

The methods based on RGB-D semantic segmentation were applied earlier in the field of computer vision. Because of the geometric consistency between the images collected by the RGB-D camera on the agent, it has a promoting effect on the deep learning semantic segmentation methods. The results of semantic segmentation can also be used to promote the positioning or closed loop detection of navigation.

#### 3.1.2. Vision Combined with LiDAR

LiDAR provide another kind of depth data in navigation environment which is more robust in light changing environment. The fusion of the visual sensor and the LiDAR information helps to improve the accuracy and stability of the navigation system.

(1) Localization

The depth data obtained by LiDAR help to improve the localization accuracy of visual navigation. Lidar-monocular visual odometry (Limo) is one example of this kind of method [34]. It proposes a depth data extraction algorithm to extract the camera track features from LiDAR data, and estimates the motion by beam adjustment (BA) from the visual key frames. Article [60] used 2D LiDAR data as assistance data and adopted a residual neural network (RES-NET) to estimate the residual between the reference depth and the actual depth, thereby completing more accurate depth estimation and enhancing the positioning accuracy. A direct visual navigation method using sparse depth point cloud of LiDAR was proposed in article [61]. The difficulty of visual sensor and LiDAR fusion comes from the fact that the resolution of the camera is much higher than that of LiDAR, so many pixels of images have no depth information. Article [62] proposed a solution to solve the problem of resolution matching between the two sensors: after calculating the geometric transformation correspondence between the two sensors, Gaussian process regression was used to interpolate the missing values. In this way, the features detected in the image can be initialized directly by using LiDAR.

(2) Map Reconstruction

LiDAR detects the boundary of the environment object well, and improves the accuracy of contour detection of the navigation system. The paper [63] drew the route and vegetation map along the river with the help of the hybrid framework of UAVs and robots. It proposed that using visual odometry combined with IMU for state estimation, and use LiDAR to detect obstacles and draw river boundaries. However, the LiDAR data may contain occluded points, which will reduce the estimation accuracy. To this end, a direct navigation method with occlusion detector and coplanar detector was proposed to solve this problem [64]. This regards the reflectivity data obtained by multi-line LiDAR as texture information, and uses visual algorithms and texture information for relocation. This information can be integrated with a high-precision map [65]. Article [66] proposed a LiDAR-enhanced SfM pipeline that jointly processes data from a rotating LiDAR and a stereo camera pair to estimate sensor motions. This approach combined with LiDAR helps to effectively reject falsely matched images and significantly improve the model’s consistency in large-scale environments.

The depth estimation of the LiDAR improves the localization accuracy of the navigation system. In addition, the fusion navigation system of LiDAR assisted the visual sensor in the edge detection of objects and the environment. However, the calibration of these two sensors automatically is still a challenge which is related to the location, posture, and the inner parameters of these two different sensors. In recent years, some researchers have put forward some innovative solutions [67,68], some of which used semantic features as indicators of calibration quality [69]. However, it is still difficult to calibrate these two sensors simultaneously in real-time practical environments.

#### 3.1.3. Vision Combined with IMU

The images obtained by the visual sensors are easily blurred when robots move rapidly. In addition, when there are too few overlapping regions between the two adjacent images for feature matching, single visual sensor navigation easily loses the motion state. Visual sensor localization combined with IMU helps to solve this problem, which is usually called visual-inertial odometry (VIO). This fusion strategy includes both spatial geometric localization and motion pose estimation [4]. In a short period of time when the visual sensor cannot obtain effective data, the data obtained by IMU can be used to maintain a good motion posture estimation [70]. The current VIO frameworks are mainly divided into two categories: loosely coupled and tightly coupled. In addition, VIO based on deep learning is also an important development direction.

(1) Loosely Coupled Methods

**Figure 1:**
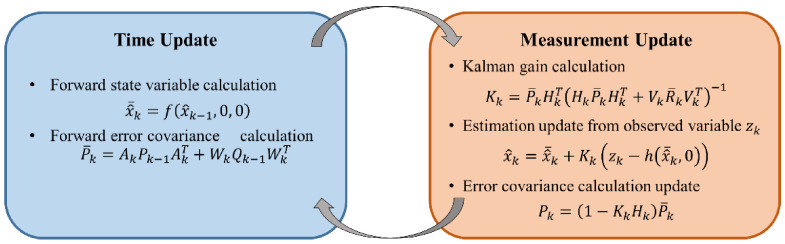
Extended Kalman filter (EKF) algorithm flow is divided into two parts: time update and measurement update. Among them, *k*−1 and *k* represent the previous state and current state respectively, ${\bar{\hat{x}}}_{k}$ is the priori estimate of current state, ${\hat{x}}_{k}$ is the posteriori estimate of the current state, $z_{k}$ is the measurement vector, *f* (·) is the nonlinear mapping equation from the previous state to the current state, *h* (·) is the nonlinear mapping equation between state and measurement, **Q** is the covariance matrix, **A** is the Jacobian matrix of the partial derivative of *f* (·) with respect to *x*, **W** is the Jacobian matrix of the partial derivative of *f* (·) with respect to noise, **H** is the Jacobian matrix of the partial derivative of *h* (·) with respect to *x*, and **V** is the Jacobian matrix of the partial derivative of *h* (·) with respect to noise.

The target of the loosely coupled method is to avoid importing image features to the state vector. The camera and the IMU perform their own motion estimation respectively, and then fuse the pose estimation. The methods usually rely on the processing of the extended Kalman filter (EKF) for fusion. The core of EKF is to establish the state prediction and update equations required for filtering [71]. The prediction equation predicts the current state based on the previous state and control amount, and the update equation indicates that the prediction result is judged by the Kalman gain. In this process, the two equations continuously update the current state (as shown in Figure 1). The loosely coupled method separately takes the image features and IMU into the observation equation to predict them separately, and finally combines the prediction results to judge the pose state.

The representatives of loosely coupled EKF fusion are the modular framework for single-sensor fusion based on an extended Kalman filter (ssf) [72] and the modular framework for multi-sensor fusion based on an extended Kalman filter (msf) [73]. The difference between them is that ssf is used for the fusion of IMU and a single sensor, while msf is used for the fusion of IMU and multiple sensors. Both of them provide relatively perfect solutions for the integration of the visual sensor and the IMU. The ssf/msf methods both aim to eliminate the accumulated errors. The visual estimation part is regarded as a black box module in the loosely coupled method. Its advantage lies in modularization: the visual motion estimation and inertial navigation motion estimation systems are two independent modules, and one sensor’s errors will not affect the other sensor. The information acquisition and processing of each module does not interfere with the other module. The loosely coupled method is easy to use due to its simple structure. However, using this kind of method, it is difficult to adjust the errors caused by visual measurement.

(2) Tightly Coupled Methods

The tightly coupled method merges the states of the camera and the IMU, and puts image features into a feature vector to construct a motion equation and observation equation. Therefore, the tightly coupled method belongs to fusion methods of feature layers. Tightly coupled methods are divided into filtering methods and nonlinear optimization methods. The representatives of filtering methods are MSCKF [36] and robust visual-inertial odometry (ROVIO) [74]. The representatives of nonlinear optimization methods are open keyframe-based visual-inertial SLAM (OKVIS) [75] and visual-inertial state monocular (VINS-mono) [76].

In the tightly coupled filtering methods represented by MSCKF and ROVIO, the feature data obtained from the visual sensor and the IMU are processed by an optimized filter, and then fused to pose estimation. This kind of method is the improvement of EKF: it based on the first-order Markov hypothesis—that is, the state of the current moment is only related to the state of the previous moment, and has nothing to do with the earlier moment before the previous one [77]. Within this method, although the calculation of the whole system will be reduced, the drift error of the previous time will accumulate to the current time. Generally speaking, the calculation of the filtering method is less than that of the optimization method.

OKVIS and VINS-mono are both nonlinear optimization methods. Most of these methods based on optimization take key frames into account. They usually save all the states of previous time, and constantly use the new observed data to correct the accumulated errors. The most ideal situation is to eliminate the errors after the detection of the closed loop. However, the disadvantage of the optimization method is that the calculation is relatively huge, and the RAM is more occupied. With the further research on the Jacobian matrix [78] and Hessian matrix [79], and the application of graph optimization tools, the calculation needed by the optimization method is greatly reduced. At present, the optimization method is the mainstream research direction of the tightly coupled method.

In total, the loosely coupled method is superior to the tightly coupled method in speed, but the fusion of different modal information is not tight enough, and the localization performance is generally not as good as the tightly coupled method. Therefore, there are more studies on tightly coupled methods than loosely coupled methods [4].

(3) Deep Learning based Coupled Methods

Recently, deep learning techniques have been widely applied in visual sensor data-dominant navigation [80]. Visual-inertial odometry with specific network (VINet) [81] and deep learning network for monocular visual-inertial odometry (DeepVIO) [82] are the representatives of these techniques. VINet uses an end-to-end trainable method for VIO which performs fusion of the data at an intermediate feature-representation level. The model combines a CNN and recurrent neural network (RNN) framework which is tailored to the task of VIO estimation. This approach is competitive with state-of-the-art traditional methods when accurate calibration data are available, and it performs better in the presence of calibration and synchronization errors. DeepVIO combines IMU data and 2D optical flow feature into the unsupervised learning framework, and uses specialized loss training to reconstruct the global camera trajectory. It employs an IMU status update scheme to improve IMU pose estimation through updating the additional gyroscope and accelerometer bias.

In particular, an important advantage of VIO deep learning is that it can correct odometry estimation errors by self-learning, saving a lot of manual debugging work [80]. Their combination has great potential to make the agent navigation more intelligent.

#### 3.1.4. Vision Combined with UWB

The UWB system provides accurate spatial coordinates and it is stable in the long term. The fusion of the UWB system and visual sensors is mostly based on monocular cameras [2,21], since the fusion of single-modal data and the UWB is structurally simple and it is easy to implement. Compared with stereo and RGB-D camera fusion, it is easier for the UWB system and the monocular camera to obtain fusion synchronization. In total, the fusion of the UWB and visual sensors includes feature-level and decision-level fusion.

(1) Feature-Level Fusion

Taking the fusion method of UWB system assisted monocular ORB-SLAM as an example [83], this method takes the independent coordinate system of the UWB localization as the global coordinate system, and transforms the coordinates of monocular ORB into the UWB coordinates by spatial transformation. The method takes all the localization and velocity errors as the state vectors of the whole monocular ORB/UWB integrated system. The localization data obtained by UWB system and monocular ORB are fused by the extended Kalman filter (EKF) algorithm, and the localization information of the fusion system is obtained by updating the state and measuring.

Deep learning methods are also involved in this aspect. Article [84] used a deep neural network to integrate UWB information into the visual positioning navigation framework. The information obtained by this framework is not only taken into navigation algorithms, but is also used to detect non-line-of-sight (NLOS) UWB measurements. Compared with the methods of using visual positioning alone, this method reduces the error by 20%.

(2) Decision-Level Fusion

This kind of method relies on the accurate spatial coordinates obtained by the UWB system to improve the loop detection performance of visual navigation methods [85]. Taking the fusion of UWB system and ORB-SLAM as an example, this method uses the ORB features and the bag-of-words model to calculate the similarity of images, and then combines the agent movement tracked by the UWB localization system to determine whether a loop occurs. This method takes the comparison of image features as the appearance similarity judgment link, and the robot motion trajectory as the spatial coordinate similarity judgment link. These two constraints jointly judge whether a loop occurs. This loop detection method makes good use of the two sensors and enhances the precision and recall of loop detection.

In general, the UWB system helps to improve the accuracy and robustness of the original visual navigation task. However, there are still some limits in use for this kind of fusion model. First, the UWB system is inconvenient in portability. Second, the stability of the UWB system depends on the correct installation and calibration of the sensors, which requires the operators to know the indoor environment in advance. Finally, the accuracy of the UWB localization will be greatly reduced in the complex indoor environment with multiple obstructions.

### 3.2. LiDAR Dominant Navigation

#### 3.2.1. LiDAR Combined with Visual Information

Although the localization accuracy of LiDAR is high and it provides real-time data, 2D LiDAR cannot recognize the color, material, and other appearance features of the object [13]. Similarly, the point cloud obtained by 3D LiDAR is relatively sparse, and the appearance of the object cannot be described well. The images collected by the visual sensor can match the visual feature points, help detect the 3D features better, and associate more semantic information.

In many cases, the LiDAR performs motion estimation through scanning matching, while the camera performs feature detection. For example, paper [86] proposed a map correction method based on laser scanning and visual loop detection, which combines a 3D LiDAR navigation with visual sensor, which performs loop detection by using the key frame technology of the bag of words model. This loop detection method enhances the performance of LiDAR navigation. The ICP algorithm can also be optimized by the combination of LiDAR and visual sensors data [87].

There are also some fusion methods that use deep learning. Article [88] proposed a global positioning algorithm called OneShot, which creatively proposes a custom neural network structure. It integrates the visual information provided by the camera into the descriptor matching of the 3D LiDAR point cloud, thereby enhancing the performance of the descriptor. Compared to using only LiDAR, fusing in the visual appearance of segments results in increased descriptor retrieval rates by 17% to 26%. Due to the high price of high-precision 3D LiDAR, some scholars have proposed methods of RGB-D or stereo cameras fusion with multi-planar 2D LiDAR to predict depth information in 3D space by DNN [89].

The deep reinforcement learning (DRL) technique has also presented its effectiveness in LiDAR combined with visual information fusion in dynamic environments. Article [90] proposed a collision avoidance algorithm, CrowdSteer, based on deep reinforcement learning, which uses 2D LiDAR as the main sensor to integrate a RGB-D camera to perceive surrounding dynamic objects and calculate collision-free speed. It uses CNN to fuse 2D LiDAR and depth image features of different dimensions, and uses proximal policy optimization (PPO) as the main training algorithm for collision avoidance and smooth trajectory formation. This method can achieve better performance in complex and occluded scenes. Article [91] proposed a multi-sensor fusion autonomous navigation method based on the Asynchronous Advantage Actor-Critic network (GA3C). This method combines the 2D depth data converted from the 3D RGB-D camera with the 2D LiDAR data, and input them into GA3C. The output is the linear velocity and angular velocity, thereby completing the robot’s motion planning. At present, this method is still in its preliminary stage, but it still can be applied in both simulation and reality. Deep learning and DRL make LiDAR and visual features more closely integrated, and it is be more adaptable to intelligent navigation work.

#### 3.2.2. LiDAR Combined with IMU

The LiDAR data are not ideal at the corner of the complex environment, which leads to the loss of details in some phases of navigation [11]. The IMU is able to compensate for the data loss of LiDAR. Some researchers use the IMU interpolation to remove the motion distortion caused by LiDAR acquisition. In general, the fusion methods of LiDAR combined with IMU can be divided into two types: feature layer fusion and decision-level fusion.

(1) Feature-level Fusion

Filtering fusion and nonlinear optimization are two kinds of the main LiDAR and IMU fusion methods which belong to feature level fusion. Filtering methods are divided into tightly coupled and loosely coupled, and optimization methods are almost tightly coupled.

In loosely coupled EKF fusion methods, when the LIDAR data are lost, EKF fusion directly compensates for the LiDAR data with the pose angle data collected by the IMU without constructing a new loss function [92]. Although the accuracy of loosely coupled EKF fusion is not particularly high, it is simple in construction and can be implemented quickly. The representative method of tightly coupled EKF fusion is robocentric LiDAR-inertial state estimator (R-LINS) [93], which uses the error state Kalman filter (ESKF) model to minimize nonlinear constraints, thereby achieving the update of the agent’s posture. It provides comparable performance to the most advanced LiDAR-inertial odometry in terms of stability and accuracy, and an order of magnitude improvement in speed.

Different from EKF fusion methods, nonlinear optimization methods use fixed lag smoothing and the marginalization of original posture to complete real-time motion estimation [94]. The typical representative methods are LiDAR-inertial odometry (LIO) [35] and its improved method: LiDAR inertial Odometry via smoothing and mapping (LIO-SAM) [95]. LIO-SAM uses a tightly coupled structure and formulates LiDAR-inertial odometry atop a factor graph. In order to improve the real-time performance of local scan matching, keyframes are selectively added to the factor graph, and sliding windows are used to marginalize old LiDAR frames.

Furthermore, feature-level methods based on deep learning are also researched. For example, article [96] proposed a method for odometry estimation using convolutional neural networks from 3D LiDAR scans. The original sparse data are encoded into 2D matrices for the training of proposed networks and for the prediction. This method can be combined with the rotation parameters obtained by IMU, while achieving high-quality odometry estimation and LiDAR data registration. This network framework shows significantly better precision in the estimation of translational motion parameters compared with LOAM, while achieving real-time performance.

Methods of feature layer fusion make full use of IMU’s advantages of collecting high-precision and small-error information in a short time, and estimate and restore the real situation of the LiDAR navigation in this period of time, so as to improve the accuracy and speed of localization and mapping. For now, tightly coupled methods have more research prospects.

(2) Decision-level Fusion

When the laser frame rate is low, the laser frame motion distortion caused by the agent motion cannot be ignored [97]. In order to eliminate the influence of motion distortion, the IMU is one of the most common assisting sensors [31]. The acquisition frequency of the IMU is very high (above 100Hz), and the IMU data are accurate in a short time period. So, when the data distortion occurs, the IMU can reflect the posture of the agent. This motion distortion removal method belongs to decision level fusion [31]. Since the IMU does not have the ability to perform visualization, the map construction of IMU and LiDAR usually relies on LiDAR mapping. In general, the methods of LiDAR and IMU when tightly coupled are better than the loosely coupled filtering methods in the localization and mapping performance.

### 3.3. UWB Combied with IMU

The UWB system has the advantage of obtaining the spatial localization information, but it is not able to obtain its motion posture. Other sensors are needed to obtain the information of motion state when the UWB acts as the dominant sensor. Fusion methods of IMU-assisted UWB were first applied to UAV indoor localization [98], and achieved outstanding results. Since the UWB and the IMU are heterogeneous, the fusion methods of them usually belong to feature layer fusion. The existing fusion methods are mainly based on the EFK methods [99].

The EKF method firstly uses the UWB system to collect and calculate the real-time coordinates of the agent, and the IMU obtains the angular acceleration and acceleration of the agent. Then, the IMU data are converted into the coordinate system where the UWB is located through the rotation matrix. Finally, the KF algorithm is used to fuse the data of the two sensors to obtain the localization information by updating of execution status and measurement [92].

In general, with the assistance of the IMU, the UWB system can reduce the difficulty of acquiring the motion state of the agent. The information collection of both of them has a high frequency, so they can easily meet the requirements of real-time synchronization. However, neither the IMU nor the UWB are able to obtain appearance information such as the shape or color of objects. It is impossible to construct a complete visual map only depending on UWB and IMU.

### 3.4. Others

There are some other situations where it is not obvious as to which sensor is the dominant one, with multi-sensor fusion based on the same kind of sensors [100], or the sensors are closely connected when three or more sensors are used at the same time. The typical example of these is visual-LiDAR odometry and mapping (V-LOAM) [20], which combines the visual sensor, LiDAR, and IMU simultaneously. It improves the VIO and the scanning matching LiDAR odometry at the same time. This method improves the performance of real-time motion estimation and point cloud registration algorithms. Some researchers used BA to align the LiDAR map with the visual map, which improves the accuracy of the whole mapping system, and enhances the robustness against environmental changes [101].

Some recent studies include the following: Article [102] proposed a fusion odometry with plane-feature tracking based on 3D LiDAR, IMU, and the camera. This method makes the sensors compensate for each other and uses a sliding window to track plane points, which makes the plane extraction efficient and robust. Article [103] proposed a tight fusion method, which takes into account the LiDAR data and the cost function of feature constraints for graph optimization, in which both LiDAR data and visual data are used to obtain the posture estimation. Article [104] proposed a compact network for absolute camera pose regression. In this approach, a 3D scene geometry-aware constraint is introduced by exploiting all available information including motion pose, depth, and image contents. For both in indoor or outdoor environments, this method has obvious performance improvement in prediction accuracy and convergence efficiency.

Due to the difficulty of technology implementation, the research on the fusion navigation method of three or more sensors has only just started. In addition to the design of fusion strategies, the collaborative processing of data is also a big challenge after multi-source information acquisition. Table 2 presents a summary of multi-sensor fusion methods for agent navigation.

## 4. Multi-Modal Datasets

To validate the performance of multiple data fusion methods, some researchers have constructed multi-modal indoor simulation datasets, and a few of them are freely available on websites [105,106,107]. These public datasets contain at least two kinds of multi-modal data, which help to validate the multi-sensor fusion methods. According to the styles of data collection, we divide multi-modal indoor simulation datasets into three types according to the data acquisition styles: the datasets acquired by the devices setup on robots, the datasets acquired by handheld devices, and those generated in virtual 3D environments.

### 4.1. Robot@Home: An Example of Datasets Acquired by The Devices Setup on Robot

**Figure 2:**
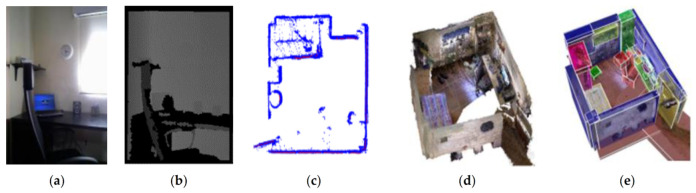
Several types of data provided by the Robot-at-Home dataset: (**a**) RGB image, (**b**) depth image, (**c**) 2D LiDAR map, (**d**) 3D room reconstruction, and (**e**) 3D room semantic reconstruction [108].

The Robot-at-Home dataset is a multi-modal dataset published by J.R. Ruiz Sarmiento in 2017, which collects multi-modal data from the home environment from the view of robot platform [108]. The Robot-at-Home dataset is a collection of raw and processed data from five domestic settings (36 rooms) compiled by a mobile robot equipped with 4 RGB-D cameras and a 2D laser scanner. In addition to the RGB-D cameras and laser scanner data, the 3D room reconstruction, the semantic information and the camera posture data are also provided (as shown in Figure 2). Therefore, the dataset is rich in contextual information of objects and rooms. The dataset contains more than 87,000 groups of data collected by mobile robots under different time nodes. The dataset samples multiple scenes to provide different perspectives of the same objects in the same scene, which shows the displacement changes of objects at different times. All modal data are correlated by the time stamp. Figure 1 presents several types of data provided by the Robot-at-Home dataset. There are some multi-modal fusion works discussed based on the Robot-at-Home dataset, such as multi-layer semantic structure analysis using RGB-D information combined with LIDAR data [109], visual odometry with the assistance of depth data [110], and path planning by the combination of depth data and LiDAR data [111], etc.

The Robot-at-Home dataset is collected in the real home environment and contains detailed semantic labels, which is also important in SLAM. There are also other multi-modal datasets acquired by the devices setup on robot, such as the MIT Stata Center Dataset [112], TUMindoor Dataset [113], Fribourg Dataset [114], and KITTI Dataset [115], etc. These datasets also provide abundant multi-modal data and are widely used in navigation research [116,117,118].

### 4.2. Microsoft 7 Scenes: An Example of Datasets Acquired by Handheld Devices

**Figure 3:**
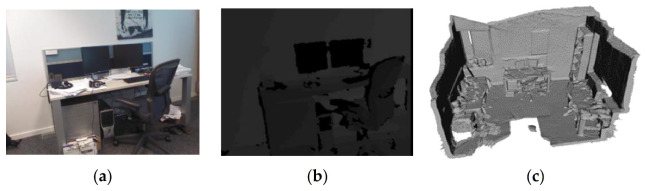
Three types of the data provided by Microsoft 7 Scenes Dataset: (**a**) RGB image, (**b**) depth image, and (**c**) TSDF volume for scene reconstruction [119].

Microsoft 7 Scenes is a multi-modal dataset released by Microsoft in 2013. The dataset contains seven scenes, and the data collection is completed by 640 × 480 resolution hand-held Kinect RGB-D camera [119]. Each scene in the dataset contains 500–1000 sets of data, and each set of data is divided into three parts (as shown in Figure 3): a RGB image, a depth image, and a 4 × 4 pose matrix from camera to world homogeneous coordinate system. For each scene, the dataset is divided into a training set and a test set. In addition, the truncated signed distance function (TSDF) volume of the scene is also provided in the dataset. Users can use these data as the basis of multi-modal information fusion, or directly reconstruct the scene using the TSDF volumes provided by the dataset. In 2013, Ben Glocker et al. proposed a camera relocalization method based on Microsoft 7 Scenes, which achieved a good result [119]. Jamie Shotton et al. proposed an approach which employs a camera relocalization method based on regression forest, which was performed on the Microsoft 7 Scenes dataset [120].

The Microsoft 7 Scenes dataset has a large quantity of samples and rich details for a single room, and the difference between adjacent samples is small. Some samples also have motion blur, which is suitable for model training related to the discrimination of scene changes. However, the data of Microsoft 7 Scenes were collected by a hand-held RGB-D camera, and thus are not suitable for research on indoor robots where viewing angles are strictly required.

### 4.3. ICL-NUIM RGB-D Benchmark Dataset: An Example of Datasets Generated in Virtual 3D Environments

**Figure 4:**
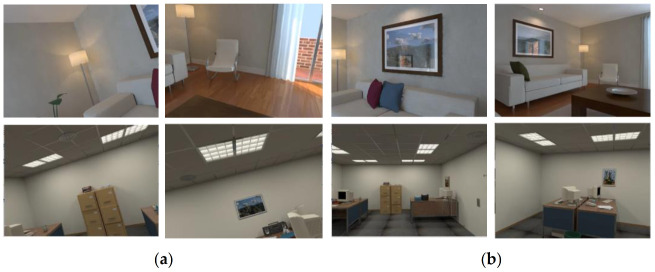
Two kinds of different view modes are designed in ICL-NUIM RGB-D Benchmark Dataset [121]. Image (**a**) shows the view mode of a variety of camera perspectives. Image (**b**) shows the view mode of a head-up camera perspective.

The ICL-NUIM RGB-D Benchmark Dataset is a dataset released by Imperial College London and the National University of Ireland in 2014. It simulates the agent motion and visual sensor posture in 3D virtual scenes [121]. The dataset contains two basic scenes: the living room and the office. There are 4601 sets of image data in each scene. Each group of data includes a RGB image, a depth image, and a 4 × 4 matrix, which describes the camera pose. For each scene, two different view modes are designed by the dataset: one is to simulate the free movement of the camera in the scene, and the collected images have a variety of perspectives (as shown in Figure 4a); the other is that the camera rotates and moves at a certain height, and the collected images only have a head-up perspective (as shown in Figure 4b). Images from different perspectives can correspond to different application scenarios. For example, multi-perspective view images may be more suitable for simulating the indoor UAV [122], while head-up perspective view images are closer to the motion state of mobile robots [123,124].

Because the data in ICL-NUIM RGB-D Benchmark Dataset are simulated 3D data, they are slightly different from real scenes. The light in the 3D scene is ideal, and the details of the collected images are rich and balanced. There are no motion blur or illumination changes in the images. These characters make the images of ICL-NUIM RGB-D unrealistic in vision, and the algorithms validated in ICL-NUIM RGB-D may not be completely effective in real practical applications.

### 4.4. Others

In addition the discussed datasets above, Table 3 presents a view for various multi-sensors agent navigation datasets, including UZH-FPV Drone Racing [125], TUM RGB-D Dataset [126], ScanNet [127], NYU V2 [128], InteriorNet [129], SceneNet RGB-D [130], and others [131,132,133,134,135,136,137,138,139,140,141,142,143,144], etc. These datasets provide the basic requirements of simulation and evaluation of multi-sensor fusion in experiments.

## 5. Discussions and Future Trends

### 5.1. Discussions

In this work, we focus on the discussions of sensors and the fusion methods of them according to their dominant functionalities in fusion. This strategy benefits the outline of each sensor, and helps to present the sensors’ advantages and disadvantages in the fusion procedure. However, because of the space limitation of this work, we did not present the sensors’ data acquisition and the data processing, such as feature extraction, sensors’ data presentations, etc., in details. Since data acquisition and process are also important factors in multi-modal information fusion, we hope that the readers will refer to the details in related references listed in this work.

In addition, in the section relating to the multi-modal dataset, the categories of the datasets were divided into three types according to the locations where the sensors are mounted. We believe that how the sensors are placed on or around the robots is critical for multi-modal sensor fusion. Although there are researchers who divided the dataset according to the number of sensors involved in the dataset, or the scale of accessible ranges for navigation, the ways that we consider the dataset from sensors’ mounting styles are useful supplements to the previous work.

### 5.2. Future Trends

With the emergence of various sensors and the continuous development of them, multi-sensor fusion will be an urgent need, which includes not only more specific algorithms, but also their applications in more practical scenes. We briefly discuss several technique trends of multi-sensor fusion navigation methods.

#### 5.2.1. Uniform Fusion Framework

The fusion methods discussed in this work are mostly bi-modal or tri-modal fusion methods and most of them are task-specific. Compared with bi-modal and tri-modal fusion methods, the fusion of more than three sensors means the whole navigation system will become more complex. There are some researchers that have already paid attention to more than three kinds of sensor fusion in robot navigation [20]. However, there is a lack of methods fusing various sensors’ information in a uniform framework, and there are problems integrating the algorithm in the robot navigation and providing a more effective fusion strategy. In the future, we need a multiple fusion framework, which is able to fuse more different modalities simultaneously in a uniform framework.

#### 5.2.2. Evaluation Methods

To evaluate a fusion model, it is necessary to establish an evaluation method for a multi-modal sensor fusion model [145]. In spite of several mature evaluation methods, such as the Monte Carlo strategy, real-time simulation, and individual calculation in the specific applications, and some SLAM evaluation methods, for instance relative pose error (RPE) [146], absolute trajectory error (ATE) [146], etc., they are not suitable to evaluate the performance of each sensor in the whole fusion system. For the multi-sensor fusion methods, we need more effective evaluation methods which are able to score not only the fusion system, but also to outline the contribution and performance of each sensor in the whole fusion process.

#### 5.2.3. On-Line Learning and Planning

With the increasing demands of practical applications, the agents are possibly required to complete some tasks that have not been learned before. These tasks need the agent to make appropriate judgments independently, which depend on the agents’ perception, on-line learning and planning in the unknown indoor environment. For example, the agent is able to learn the name and localization of unknown objects in the scene, so as to effectively feedback the tasks and instructions given by users. The related techniques include interactive learning, knowledge transfer leaning, etc., from the multi-modal fusion data. With these methods, the agents are hoped to obtain the ability to correct itself with continuous learning in continuous environments, simulate human thinking and make decisions in dynamic environments.

## 6. Conclusions

Multi sensor fusion has become an important research direction in mobile agent navigation. We introduce the mainstream techniques of multi-sensor fusion methods for mobile agents’ indoor autonomous navigation in this work, including: single-sensor navigation methods, multi-sensor fusion navigation methods, some well-recognized multi-modal datasets, and the trend of future development. We believe with the increasing demand for human–computer interaction, mobile agents with multi-sensor fusion will be more intelligent and interactive in the future.

## Figures and Tables

**Table 1:** List of single-sensor navigation methods.

Sensor	Methods	Environments	Advantages	Disadvantages
Monocular Camera	ORB-SLAM [2] LSD-SLAM [24] SVO [25]	Small/medium indoors	Robust and versatile	Sensitive to illumination changes
Stereo Camera	ORB-SLAM 2 [21] RTAB-MAP [22]	Small/medium indoors	Higher accuracy but fine calculation	Sensitive to illumination changes
RGB-D Camera	RGBD-SLAM-V2 [27] DVO-SLAM [28] RTAB-MAP [22]	Small/medium indoors	Acquisitively of depth information	Significant noise
LiDAR	GMapping [29] Cartographer [32] IMLS-SLAM [30] LOAM [31]	Both indoors and outdoors	High measurement accuracy	Insufficient in supporting object detection
IMU	Combined with LiDAR: LIO [35] Combined with camera: MSCKF [36]	Only measure itself	Accurate in a short time	Unavoidable and obvious cumulative errors
UWB System	Ranging algorithm: TOF [39], AOA [40], TOA [40] and TDOA [37]	Indoors	High location accuracy	Insufficient flexibility in applications
Wi-Fi, Bluetooth, ZigBee, infrared, ultrasonic, etc.	Distance measurement method which is similar to UWB system [6,7,8,9,10]	Mainly indoors	High applicability of visually impaired environments	Insufficient location accuracy and vulnerable to occlusion interference

**Table 2:** Summary of multi-sensor fusion navigation methods.

Dominant Sensor	Assisted Sensor	Related Works
Visual Sensor	Depth	Motion Estimation Methods: Feature points’ matching (PnP [46]/ICP [47]) Optical flow (LK optical flow [54], based on deep learning [56,57]) Direct method (sparse/dense/semi-dense [27,28]) Semantic SLAM based on deep architectures [58,59]
	LiDAR	Fusion location in feature level [34,60,61,62] Assistance of edge detection of visual detection [63,64,65]
	IMU	Loosely Coupled (ssf [72]/msf [73]) Tightly Coupled (Filtering Methods: MSCKF [36]/ROVIO [74] and Optimization Methods: OKVIS [75]/VINS-mono [76]) Deep learning based coupled methods [81,82]
	UWB System	Feature-level fusion (EKF fusion [83], based on deep learning [84]) Decision-level fusion (Fusion loop detection [85])
LiDAR	Visual Sensor	Fusion loop detection [86,87] Optimization of 3D LiDAR point cloud [13] Based on deep learning [88,89] Based on DRL [90,91]
	IMU	Feature-level fusion (based on EKF [92,93], nonlinear optimization method [35,94,95] Decision-level fusion (Fusion motion estimation [31,97])
UWB System	IMU	EKF fusion location [98,99]
Others		No dominant sensor (V-LOAM [20], and others [102,103,104])

**Table 3:** A List of Multi-modal Datasets.

Category	Representatives	Modalities	Related Works	Year
Devices Setup on Robots	Robot@Home [108]	RGB Depth LiDAR IMU	Semantic Structure Analysis [109], LiDAR Odometery [110], Path Planning [111], etc.	2017
	UZH-FPV Drone Racing [125]	RGB Depth IMU	Visual Feature Tracking [131], UAV Navigation [132], etc.	2019
	TUM RGB-D Dataset [126]	RGB Depth Camera Posture	Visual SLAM [2,21,24,25,28]	2012
Datasets Collected by Hand	Microsoft 7 Scenes [119]	RGB Depth Camera Posture	Camera Relocation [119,120], 3D Reconstruction [133], RGB-D SLAM [134], etc.	2013
	ScanNet [127]	RGB Depth Camera Posture Semantic Labels	Visual Feature Extraction [135], 3D Point Cloud [136], etc.	2017
	NYU V2 [128]	RGB Depth Semantic Labels	Semantic Segmentation [137,138,139]	2012
Datasets of 3D Virtual Scenes	ICL-NUIM RGB-D Benchmark [121]	RGB Depth Camera Posture	UAV Pose Estimation [122], RGB-D SLAM [123,124], etc.	2014
	InteriorNet [129]	RGB Depth Semantic Labels	Semantic Dataset [140], RGB-D SLAM [141], Computer Vision [142], etc.	2018
	SceneNet RGB-D [130]	RGB Depth Camera Posture, Semantic Labels	Semantic Dataset [143], Fusion Pose Estimation [144], etc.	2016

## References

[1] Bresson G., Alsayed Z., Yu L., Glaser S. (2017). Simultaneous localization and mapping: A survey of current trends in autonomous driving. IEEE Trans. Intell. Veh..

[2] Mur-Artal R., Montiel J.M.M., Tardos J.D. (2015). ORB-SLAM: A versatile and accurate monocular SLAM system. IEEE Trans. Robot..

[3] Kohlbrecher S., Von Stryk O., Meyer J., Klingauf U. A flexible and scalable SLAM system with full 3D motion estimation. Proceedings of the 2011 IEEE International Symposium on Safety, Security, and Rescue Robotics.

[4] Huang G. Visual-inertial navigation: A concise review. Proceedings of the 2019 International Conference on Robotics and Automation (ICRA).

[5] Liu L., Liu Z., Barrowes B.E. (2011). Through-wall bio-radiolocation with UWB impulse radar: Observation, simulation and signal extraction. IEEE J. Sel. Top. Appl. Earth Obs. Remote Sens..

[6] He S., Chan S.-H.G. (2015). Wi-Fi Fingerprint-based indoor positioning: Recent advances and comparisons. IEEE Commun. Surv. Tutor..

[7] Faragher R., Harle R. (2015). Location fingerprinting with bluetooth low energy beacons. IEEE J. Sel. Areas Commun..

[8] Kaemarungsi K., Ranron R., Pongsoon P. Study of received signal strength indication in ZigBee location cluster for indoor localization. Proceedings of the 2013 10th International Conference on Electrical Engineering/Electronics, Computer, Telecommunications and Information Technology.

[9] Shin Y.-S., Kim A. (2019). Sparse depth enhanced direct thermal-infrared SLAM beyond the visible spectrum. IEEE Robot. Autom. Lett..

[10] Freye C., Bendicks C., Lilienblum E., Al-Hamadi A. (2014). Multiple camera approach for SLAM based ultrasonic tank roof inspection. Image Analysis and Recognition, Proceedings of the ICIAR 2014, Vilamoura, Portugal, 22–24 October 2014.

[11] Cadena C., Carlone L., Carrillo H., Latif Y., Scaramuzza D., Neira J., Reid I.D., Leonard J.J. (2016). Past, present, and future of simultaneous localization and mapping: Toward the robust-perception age. IEEE Trans. Robot..

[12] Davison A.J. Davison real-time simultaneous localisation and mapping with a single camera. Proceedings of the Ninth IEEE International Conference on Computer Vision.

[13] Debeunne C., Vivet D. (2020). A review of visual-LiDAR fusion based simultaneous localization and mapping. Sensors.

[14] Kunhoth J., Karkar A., Al-Maadeed S., Al-Ali A. (2020). Indoor positioning and wayfinding systems: A survey. Hum. Cent. Comput. Inf. Sci..

[15] Otero R., Lagüela S., Garrido I., Arias P. (2020). Mobile indoor mapping technologies: A review. Autom. Constr..

[16] Maehara Y., Saito S. (2007). The relationship between processing and storage in working memory span: Not two sides of the same coin. J. Mem. Lang..

[17] Town C. (2006). Multi-sensory and multi-modal fusion for sentient computing. Int. J. Comput. Vis..

[18] Yang M., Tao J. (2019). A review on data fusion methods in multimodal human computer dialog. Virtual Real. Intell. Hardw..

[19] Graeter J., Wilczynski A., Lauer M. Limo: LiDAR-monocular visual odometry. Proceedings of the 2018 IEEE/RSJ International Conference on Intelligent Robots and Systems (IROS).

[20] Ji Z., Singh S. Visual-LiDAR odometry and mapping: Low-drift, robust, and fast. Proceedings of the IEEE International Conference on Robotics & Automation.

[21] Mur-Artal R., Tardos J.D. (2017). ORB-SLAM2: An open-source SLAM system for monocular, stereo, and RGB-D cameras. IEEE Trans. Robot..

[22] Labbé M., Michaud F. (2018). RTAB-map as an open-source LiDAR and visual simultaneous localization and mapping library for large-scale and long-term online operation: Labb and michaud. J. Field Robot..

[23] Klein G., Murray D. Parallel tracking and mapping for small ar workspaces. Proceedings of the 2007 6th IEEE and ACM International Symposium on Mixed and Augmented Reality.

[24] Engel J., Schps T., Cremers D. (2014). LSD-SLAM: Large-scale direct monocular slam. Proceedings of the 2014 European Conference on Computer Vision.

[25] Forster C., Zhang Z., Gassner M., Werlberger M., Scaramuzza D. (2016). SVO: Semidirect visual odometry for monocular and multicamera systems. IEEE Trans. Robot..

[26] Taketomi T., Uchiyama H., Ikeda S. (2017). Visual SLAM algorithms: A survey from 2010 to 2016. IPSJ Trans. Comput. Vis. Appl..

[27] Endres F., Hess J., Sturm J., Cremers D., Burgard W. (2014). 3-D mapping with an RGB-D camera. IEEE Trans. Robot..

[28] Kerl C., Sturm J., Cremers D. Dense visual SLAM for RGB-D cameras. Proceedings of the 2013 IEEE/RSJ International Conference on Intelligent Robots and Systems.

[29] Grisetti G., Stachniss C., Burgard W. (2007). Improved techniques for grid mapping with Rao-Blackwellized particle filters. IEEE Trans. Robot..

[30] Deschaud J.E. IMLS-SLAM: Scan-to-model matching based on 3d data. Proceedings of the IEEE International Conference on Robotics and Automation.

[31] Zhang J., Singh S. LOAM: LiDAR odometry and mapping in real-time. Proceedings of the Robotics: Science and Systems.

[32] Hess W., Kohler D., Rapp H., Andor D. Real-time loop closure in 2D LiDAR SLAM. Proceedings of the 2016 IEEE International Conference on Robotics and Automation (ICRA).

[33] Zhang R., Hoflinger F., Reindl L. (2013). Inertial sensor based indoor localization and monitoring system for emergency responders. IEEE Sensors J..

[34] Gui J., Gu D., Wang S., Hu H. (2015). A review of visual inertial odometry from filtering and optimisation perspectives. Adv. Robot..

[35] Ye H., Chen Y., Liu M. Tightly coupled 3D LiDAR inertial odometry and mapping. Proceedings of the 2019 International Conference on Robotics and Automation (ICRA).

[36] Mourikis A.I., Roumeliotis S.I. A Multi-state constraint kalman filter for vision-aided inertial navigation. Proceedings of the 2007 IEEE International Conference on Robotics and Automation.

[37] Young D.P., Keller C.M., Bliss D.W., Forsythe K.W. Ultra-wideband (UWB) transmitter location using time difference of arrival (TDOA) techniques. Proceedings of the Thrity-Seventh Asilomar Conference on Signals, Systems & Computers 2003.

[38] Porcino D., Hirt W. (2003). Ultra-wideband radio technology: Potential and challenges ahead. IEEE Commun. Mag..

[39] Despaux F., Bossche A.V.D., Jaffrès-Runser K., Val T. (2018). N-TWR: An accurate time-of-flight-based N-ary ranging protocol for Ultra-Wide band. Ad Hoc Netw..

[40] Iwakiri N., Kobayashi T. Joint TOA and AOA estimation of UWB signal using time domain smoothing. Proceedings of the 2007 2nd International Symposium on Wireless Pervasive Computing.

[41] Al-Madani B., Orujov F., Maskeliūnas R., Damaševičius R., Venčkauskas A. (2019). Fuzzy logic type-2 based wireless indoor localization system for navigation of visually impaired people in buildings. Sensors.

[42] Orujov F., Maskeliūnas R., Damaeviius R., Wei W., Li Y. (2018). Smartphone based intelligent indoor positioning using fuzzy logic. Future Gener. Comput. Syst..

[43] Wietrzykowski J., Skrzypczynski P. A fast and practical method of indoor localization for resource-constrained devices with limited sensing. Proceedings of the 2020 IEEE International Conference on Robotics and Automation (ICRA).

[44] Guo Y., Wang H., Hu Q., Liu H., Liu L., Bennamoun M. (2020). Deep learning for 3D point clouds: A survey. IEEE Trans. Pattern Anal. Mach. Intell..

[45] Lowe D.G. (2004). Distinctive image features from scale-invariant keypoints. Int. J. Comput. Vis..

[46] Li S., Xu C., Xie M. (2012). A robust O(n) solution to the perspective-n-point problem. IEEE Trans. Pattern Anal. Mach. Intell..

[47] Besl P.J., McKay N.D. (1992). A method for registration of 3-D shapes. IEEE Trans. Pattern Anal. Mach. Intell..

[48] Pomerleau F., Colas F., Siegwart R. (2015). A review of point cloud registration algorithms for mobile robotics. Found. Trends Robot..

[49] Barone F., Marrazzo M., Oton C.J. (2020). Camera calibration with weighted direct linear transformation and anisotropic uncertainties of image control points. Sensors.

[50] Li T., Pei L., Xiang Y., Wu Q., Xia S., Tao L., Yu W. (2021). P3-LOAM: PPP/LiDAR loosely coupled SLAM with accurate covariance estimation and robust RAIM in urban canyon environment. IEEE Sens. J..

[51] Zhang H., Ye C. DUI-VIO: Depth uncertainty incorporated visual inertial odometry based on an RGB-D camera. Proceedings of the 2020 IEEE/RSJ International Conference on Intelligent Robots and Systems (IROS).

[52] Sorkine O. (2009). Least-squares rigid motion using SVD. Tech. Notes.

[53] Triggs B., McLauchlan P.F., Hartley R.I., Fitzgibbon A.W. (1999). Bundle adjustment—A modern synthesis. Vision Algorithms: Theory and Practice, Proceedings of the International Workshop on Vision Algorithms, Corfu, Greece, 21–22 September 1999.

[54] Bouguet J.Y. (2001). Pyramidal implementation of the affine Lucas Kanade feature tracker description of the algorithm. Intel Corp..

[55] Ojala T., Pietikainen M., Maenpaa T. (2002). Multiresolution gray-scale and rotation invariant texture classification with local binary patterns. IEEE Trans. Pattern Anal. Mach. Intell..

[56] Zhang T., Zhang H., Nakamura Y., Yang L., Zhang L. Flowfusion: Dynamic dense RGB-D SLAM based on optical flow. Proceedings of the 2020 IEEE International Conference on Robotics and Automation (ICRA).

[57] Xu J., Ranftl R., Koltun V. Accurate optical flow via direct cost volume processing. Proceedings of the 2017 IEEE Conference on Computer Vision and Pattern Recognition (CVPR).

[58] Ma L., Stuckler J., Kerl C., Cremers D. Multi-view deep learning for consistent semantic mapping with RGB-D cameras. Proceedings of the 2017 IEEE/RSJ International Conference on Intelligent Robots and Systems (IROS).

[59] Qi X., Liao R., Jia J., Fidler S., Urtasun R. In 3D graph neural networks for RGBD semantic segmentation. Proceedings of the 2017 IEEE International Conference on Computer Vision (ICCV).

[60] Liao Y., Huang L., Wang Y., Kodagoda S., Yu Y., Liu Y. Parse geometry from a line: Monocular depth estimation with partial laser observation. Proceedings of the 2017 IEEE International Conference on Robotics and Automation (ICRA).

[61] Shin Y.S., Park Y.S., Kim A. Direct visual SLAM using sparse depth for camera-LiDAR system. Proceedings of the 2018 International Conference on Robotics and Automation.

[62] De Silva V., Roche J., Kondoz A. (2017). Fusion of LiDAR and camera sensor data for environment sensing in driverless vehicles. arXiv.

[63] Scherer S., Rehder J., Achar S., Cover H., Chambers A., Nuske S., Singh S. (2012). River mapping from a flying robot: State estimation, river detection, and obstacle mapping. Auton. Robot..

[64] Huang K., Xiao J., Stachniss C. Accurate direct visual-laser odometry with explicit occlusion handling and plane detection. Proceedings of the 2019 International Conference on Robotics and Automation (ICRA).

[65] Pascoe G., Maddern W., Newman P. Direct visual localisation and calibration for road vehicles in changing city environments. Proceedings of the 2015 IEEE International Conference on Computer Vision Workshop (ICCVW).

[66] Zhen W., Hu Y., Yu H., Scherer S. LiDAR-enhanced structure-from-motion. Proceedings of the 2020 IEEE International Conference on Robotics and Automation (ICRA).

[67] Park C., Moghadam P., Kim S., Sridharan S., Fookes C. (2020). Spatiotemporal camera-LiDAR calibration: A targetless and structureless approach. IEEE Robot. Autom. Lett..

[68] Kummerle J., Kuhner T. Unified intrinsic and extrinsic camera and LiDAR calibration under uncertainties. Proceedings of the 2020 IEEE International Conference on Robotics and Automation (ICRA) Paris.

[69] Zhu Y., Li C., Zhang Y. Online camera-LiDAR calibration with sensor semantic information. Proceedings of the 2020 IEEE International Conference on Robotics and Automation (ICRA).

[70] Delmerico J., Scaramuzza D. A benchmark comparison of monocular visual-inertial odometry algorithms for flying robots. Proceedings of the 2018 IEEE International Conference on Robotics and Automation (ICRA).

[71] Sun S.-L., Deng Z.-L. (2004). Multi-sensor optimal information fusion Kalman filter. Automatica.

[72] Weiss S., Siegwart R. Real-time metric state estimation for modular vision-inertial systems. Proceedings of the 2011 IEEE International Conference on Robotics and Automation.

[73] Lynen S., Achtelik M.W., Weiss S., Chli M., Siegwart R. A robust and modular multi-sensor fusion approach applied to MAV navigation. Proceedings of the 2013 IEEE/RSJ International Conference on Intelligent Robots and Systems.

[74] Bloesch M., Omari S., Hutter M., Siegwart R. Robust visual inertial odometry using a direct EKF-based approach. Proceedings of the 2015 IEEE/RSJ International Conference on Intelligent Robots and Systems (IROS).

[75] Leutenegger S., Lynen S., Bosse M., Siegwart R., Furgale P. (2015). Keyframe-based visual–inertial odometry using nonlinear optimization. Int. J. Robot. Res..

[76] Qin T., Li P., Shen S. (2018). VINS-Mono: A robust and versatile monocular visual-inertial state estimator. IEEE Trans. Robot..

[77] Li M., Mourikis A.I. (2013). High-precision, consistent EKF-based visual-inertial odometry. Int. J. Robot. Res..

[78] Kim C., Sakthivel R., Chung W.K. (2008). Unscented FastSLAM: A robust and efficient solution to the SLAM problem. IEEE Trans. Robot..

[79] Thrun S., Montemerlo M. (2006). The graph SLAM algorithm with applications to large-scale mapping of urban structures. Int. J. Robot. Res..

[80] Chen C., Wang B., Lu C.X., Trigoni N., Markham A. (2020). A survey on deep learning for localization and mapping: Towards the age of spatial machine intelligence. arXiv.

[81] Clark R., Wang S., Wen H., Markham A., Trigoni N. Vinet: Visual-inertial odometry as a sequence-to-sequence learning problem. Proceedings of the 2017 AAAI Conference on Artificial Intelligence.

[82] Han L., Lin Y., Du G., Lian S. DeepVIO: Self-supervised deep learning of monocular visual inertial odometry using 3D geometric constraints. Proceedings of the 2019 IEEE/RSJ International Conference on Intelligent Robots and Systems (IROS).

[83] Benini A., Mancini A., Longhi S. (2013). An IMU/UWB/vision-based extended Kalman filter for mini-UAV localization in indoor environment using 802.15.4a wireless sensor network. J. Intell. Robot. Syst..

[84] Masiero A., Perakis H., Gabela J., Toth C., Gikas V., Retscher G., Goel S., Kealy A., Koppányi Z., Błaszczak-Bak W. (2020). Indoor navigation and mapping: Performance analysis of UWB-based platform positioning. Int. Arch. Photogramm. Remote Sens. Spat. Inf. Sci..

[85] Queralta J.P., Almansa C.M., Schiano F., Floreano D., Westerlund T. UWB-based system for UAV localization in GNSS-denied environments: Characterization and dataset. Proceedings of the 2020 IEEE/RSJ International Conference on Intelligent Robots and Systems (IROS).

[86] Zhu Z., Yang S., Dai H., Li F. (2018). Loop detection and correction of 3D laser-based SLAM with visual information. Proceedings of the Proceedings of the 31st International Conference on Computer Animation and Social Agents—CASA 2018.

[87] Pandey G., Mcbride J.R., Savarese S., Eustice R.M. Visually bootstrapped generalized ICP. Proceedings of the IEEE International Conference on Robotics & Automation.

[88] Ratz S., Dymczyk M., Siegwart R., Dubé R. Oneshot global localization: Instant LiDAR-visual pose estimation. Proceedings of the 2020 IEEE International Conference on Robotics and Automation (ICRA).

[89] Zhang J., Ramanagopal M.S., Vasudevan R., Johnson-Roberson M. LiStereo: Generate dense depth maps from LiDAR and Stereo Imagery. Proceedings of the 2020 IEEE International Conference on Robotics and Automation (ICRA).

[90] Liang J., Patel U., Sathyamoorthy A.J., Manocha D. (2020). Realtime collision avoidance for mobile robots in dense crowds using implicit multi-sensor fusion and deep reinforcement learning. arXiv.

[91] Surmann H., Jestel C., Marchel R., Musberg F., Elhadj H., Ardani M. (2020). Deep reinforcement learning for real autonomous mobile robot navigation in indoor environments. arXiv.

[92] Hol J.D., Dijkstra F., Luinge H., Schon T.B. Tightly coupled UWB/IMU pose estimation. Proceedings of the 2009 IEEE International Conference on Ultra-Wideband.

[93] Qin C., Ye H., Pranata C., Han J., Zhang S., Liu M. (2019). R-lins: A robocentric LiDAR-inertial state estimator for robust and efficient navigation. arXiv.

[94] Moore J.B. (1973). Discrete-time fixed-lag smoothing algorithms. Automatica.

[95] Shan T., Englot B., Meyers D., Wang W., Rus D. (2020). Lio-sam: Tightly-coupled LiDAR inertial odometry via smoothing and mapping. arXiv.

[96] Velas M., Spanel M., Hradis M., Herout A. CNN for IMU assisted odometry estimation using velodyne LiDAR. Proceedings of the 2018 IEEE International Conference on Autonomous Robot Systems and Competitions (ICARSC).

[97] Le Gentil C., Vidal-Calleja T., Huang S. 3D LiDAR-IMU calibration based on upsampled preintegrated measurements for motion distortion correction. Proceedings of the 2018 IEEE International Conference on Robotics and Automation (ICRA).

[98] Mueller M.W., Hamer M., D’Andrea R. Fusing ultra-wideband range measurements with accelerometers and rate gyroscopes for quadrocopter state estimation. Proceedings of the 2015 IEEE International Conference on Robotics and Automation (ICRA).

[99] Corrales J.A., Candelas F.A., Torres F. Hybrid tracking of human operators using IMU/UWB data fusion by a Kalman filter. Proceedings of the 3rd International Conference on Intelligent Information Processing; Association for Computing Machinery (ACM).

[100] Zhang M., Xu X., Chen Y., Li M. (2020). A Lightweight and accurate localization algorithm using multiple inertial measurement units. IEEE Robot. Autom. Lett..

[101] Ding X., Wang Y., Li D., Tang L., Yin H., Xiong R. Laser map aided visual inertial localization in changing environment. Proceedings of the 2018 IEEE/RSJ International Conference on Intelligent Robots and Systems (IROS).

[102] Zuo X., Yang Y., Geneva P., Lv J., Liu Y., Huang G., Pollefeys M. (2020). Lic-fusion 2.0: LiDAR-inertial-camera odometry with sliding-window plane-feature tracking. arXiv.

[103] Jiang G., Yin L., Jin S., Tian C., Ma X., Ou Y. (2019). A simultaneous localization and mapping (SLAM) framework for 2.5D map building based on low-cost LiDAR and vision fusion. Appl. Sci..

[104] Tian M., Nie Q., Shen H. 3D scene geometry-aware constraint for camera localization with deep learning. Proceedings of the 2020 IEEE International Conference on Robotics and Automation (ICRA).

[105] Robot@Home Dataset. http://mapir.isa.uma.es/mapirwebsite/index.php/mapir-downloads/203-robot-at-home-dataset.

[106] Rgb-D Dataset 7-Scenes—Microsoft Research. https://www.microsoft.com/en-us/research/project/rgb-d-dataset-7-scenes/.

[107] Imperial College London ICL-NUIM RGB-D Benchmark Dataset. http://www.doc.ic.ac.uk/~ahanda/VaFRIC/iclnuim.html.

[108] Ruiz-Sarmiento J.R., Galindo C., Gonzalez-Jimenez J. (2017). Robot@Home, a robotic dataset for semantic mapping of home environments. Int. J. Robot. Res..

[109] Ruiz-Sarmiento J.R., Galindo C., Gonzalez-Jimenez J. (2017). Building multiversal semantic maps for mobile robot operation. Knowl. Based Syst..

[110] Mariano J., Javier M., Manuel L.A., Javier G.J. (2018). Robust planar odometry based on symmetric range flow and multiscan alignment. IEEE Trans. Robot..

[111] Moreno F.-A., Monroy J., Ruiz-Sarmiento J.-R., Galindo C., Gonzalez-Jimenez J. (2019). Automatic waypoint generation to improve robot navigation through narrow spaces. Sensors.

[112] Fallon M., Johannsson H., Kaess M., Leonard J.J. (2013). The MIT Stata Center dataset. Int. J. Robot. Res..

[113] Huitl R., Schroth G., Hilsenbeck S., Schweiger F., Steinbach E. TUMindoor: An extensive image and point cloud dataset for visual indoor localization and mapping. Proceedings of the 2012 19th IEEE International Conference on Image Processing.

[114] Blanco-Claraco J.-L., Moreno-Dueñas F.-Á., González-Jiménez J. (2014). The Málaga urban dataset: High-rate stereo and LiDAR in a realistic urban scenario. Int. J. Robot. Res..

[115] Geiger A., Lenz P., Stiller C., Urtasun R. (2013). Vision meets robotics: The KITTI dataset. Int. J. Robot. Res..

[116] Rusli I., Trilaksono B.R., Adiprawita W. (2020). RoomSLAM: Simultaneous localization and mapping with objects and indoor layout structure. IEEE Access.

[117] Nikoohemat S., Diakité A.A., Zlatanova S., Vosselman G. (2020). Indoor 3D reconstruction from point clouds for optimal routing in complex buildings to support disaster management. Autom. Constr..

[118] Feng D., Haase-Schutz C., Rosenbaum L., Hertlein H., Glaser C., Timm F., Wiesbeck W., Dietmayer K. (2020). Deep multi-modal object detection and semantic segmentation for autonomous driving: Datasets, methods, and challenges. IEEE Trans. Intell. Transp. Syst..

[119] Glocker B., Izadi S., Shotton J., Criminisi A. Real-time RGB-D camera relocalization. Proceedings of the IEEE International Symposium on Mixed & Augmented Reality.

[120] Shotton J., Glocker B., Zach C., Izadi S., Criminisi A., FitzGibbon A. Scene coordinate regression forests for camera relocalization in RGB-D images. Proceedings of the 2013 IEEE Conference on Computer Vision and Pattern Recognition.

[121] Handa A., Whelan T., McDonald J., Davison A.J. A benchmark for RGB-D visual odometry, 3D reconstruction and SLAM. Proceedings of the 2014 IEEE International Conference on Robotics and Automation (ICRA).

[122] Shetty A., Gao G.X. UAV pose estimation using cross-view geolocalization with satellite imagery. Proceedings of the 2019 International Conference on Robotics and Automation (ICRA).

[123] Whelan T., Leutenegger S., Salas-Moreno R.F., Glocker B., Davison A.J. Elasticfusion: Dense SLAM without a pose graph. Proceedings of the Robotics: Science & Systems 2015.

[124] Tateno K., Tombari F., Laina I., Navab N. CNN-SLAM: Real-time dense monocular SLAM with learned depth prediction. Proceedings of the 2017 IEEE Conference on Computer Vision and Pattern Recognition (CVPR).

[125] Delmerico J., Cieslewski T., Rebecq H., Faessler M., Scaramuzza D. Are we ready for autonomous drone racing? The UZH-FPV drone racing dataset. Proceedings of the 2019 International Conference on Robotics and Automation (ICRA).

[126] Sturm J., Engelhard N., Endres F., Burgard W., Cremers D. A benchmark for the evaluation of RGB-D SLAM systems. Proceedings of the 2012 IEEE/RSJ International Conference on Intelligent Robots and Systems.

[127] Dai A., Chang A.X., Savva M., Halber M., Funkhouser T., Niessner M. ScanNet: Richly-annotated 3D reconstructions of indoor scenes. Proceedings of the 2017 IEEE Conference on Computer Vision and Pattern Recognition (CVPR).

[128] Silberman N., Hoiem D., Kohli P., Fergus R. Indoor segmentation and support inference from RGBD images. Proceedings of the 2012 European Conference on Computer Vision (ECCV).

[129] Li W., Saeedi S., McCormac J., Clark R., Tzoumanikas D., Ye Q., Huang Y., Tang R., Leutenegger S. (2018). Interiornet: Mega-scale multi-sensor photo-realistic indoor scenes dataset. arXiv.

[130] McCormac J., Handa A., Leutenegger S., Davison A.J. (2016). Scenenet RGB-D: 5m photorealistic images of synthetic indoor trajectories with ground truth. arXiv.

[131] Gehrig D., Rebecq H., Gallego G., Scaramuzza D. (2020). EKLT: Asynchronous photometric feature tracking using events and frames. Int. J. Comput. Vis..

[132] Rodriguez-Gomez J., Eguiluz A.G., Dios J.M.-D., Ollero A. Asynchronous event-based clustering and tracking for intrusion monitoring in UAS. Proceedings of the 2020 IEEE International Conference on Robotics and Automation (ICRA).

[133] Leibe B., Matas J., Sebe N., Welling M. Real-time Large-Scale Dense 3D Reconstruction with Loop Closure. Proceedings of the 2016 European Conference on Computer Vision (ECCV).

[134] Taira H., Okutomi M., Sattler T., Cimpoi M., Pollefeys M., Sivic J., Pajdla T., Torii A. (2019). InLoc: Indoor visual localization with dense matching and view synthesis. IEEE Trans. Pattern Anal. Mach. Intell..

[135] Qi C.R., Yi L., Su H., Guibas L.J. (2017). Pointnet++: Deep hierarchical feature learning on point sets in a metric space. arXiv.

[136] Li Y., Bu R., Sun M., Wu W., Di X., Chen B. (2018). Pointcnn: Convolution on x-transformed points. arXiv.

[137] Long J., Shelhamer E., Darrell T. Fully convolutional networks for semantic segmentation. Proceedings of the IEEE Conference on Computer Vision and Pattern Recognition.

[138] Badrinarayanan V., Kendall A., Cipolla R. (2017). SegNet: A deep convolutional encoder-decoder architecture for image segmentation. IEEE Trans. Pattern Anal. Mach. Intell..

[139] Eigen D., Fergus R. Predicting depth, surface normals and semantic labels with a common multi-scale convolutional architecture. Proceedings of the 2015 IEEE International Conference on Computer Vision (ICCV).

[140] Behley J., Garbade M., Milioto A., Quenzel J., Behnke S., Stachniss C., Gall J. SemanticKITTI: A dataset for semantic scene understanding of LiDAR Sequences. Proceedings of the 2019 IEEE/CVF International Conference on Computer Vision (ICCV).

[141] Xu B., Li W., Tzoumanikas D., Bloesch M., Davison A., Leutenegger S. MID-Fusion: Octree-based object-level multi-instance dynamic SLAM. Proceedings of the 2019 International Conference on Robotics and Automation (ICRA).

[142] Zhang S., He F., Ren W., Yao J. (2018). Joint learning of image detail and transmission map for single image dehazing. Vis. Comput..

[143] Armeni I., Sax S., Zamir A.R., Savarese S. (2017). Joint 2D–3D-semantic data for indoor scene understanding. arXiv.

[144] Tremblay J., To T., Sundaralingam B., Xiang Y., Fox D., Birchfield S. (2018). Deep object pose estimation for semantic robotic grasping of household objects. arXiv.

[145] Bujanca M., Gafton P., Saeedi S., Nisbet A., Bodin B., O’Boyle M.F.P., Davison A.J., Kelly P.H.J., Riley G., Lennox B. SLAMbench 3.0: Systematic automated reproducible evaluation of slam systems for robot vision challenges and scene understanding. Proceedings of the 2019 International Conference on Robotics and Automation.

[146] Zhang Z., Scaramuzza D. A tutorial on quantitative trajectory evaluation for visual(-inertial) odometry. Proceedings of the 2018 IEEE/RSJ International Conference on Intelligent Robots and Systems (IROS).

